# COVID-19 Pandemic Impacts on STRESS, PTSD, and Prefrontal Cortical Thickness in Pre-Pandemic Trauma Survivors

**DOI:** 10.3390/jpm15040127

**Published:** 2025-03-26

**Authors:** Sharad Chandra, Atheer Amer, Chia-Hao Shih, Qin Shao, Xin Wang, Hong Xie

**Affiliations:** 1 Department of Neurosciences and Psychiatry, University of Toledo College of Medicine and Life Sciences, Toledo, OH 43614, USA; 2 Department of Emergency Medicine, University of Toledo College of Medicine and Life Sciences, Toledo, OH 43614, USA; 3 Department of Mathematics and Statistics, University of Toledo, Toledo, OH 43606, USA

**Keywords:** COVID-19 pandemic, trauma survivor, cortical thickness, prefrontal cortex, structural MRI

## Abstract

**Background/Objectives**: The COVID-19 pandemic increased psychiatric symptoms in patients with pre-pandemic mental health conditions. However, the effects of pandemic on the brain, stress, and mental illness remain largely conjectural. Our objective was to examine how the pandemic affected prefrontal cortical thicknesses (CTs), stress, and PTSD symptoms in people with pre-pandemic trauma histories. **Methods**: Fifty-one survivors from a pre-pandemic trauma study who had completed a pre-pandemic PTSD Checklist-5 (PCL) to assess PTSD symptoms and a sMRI scan to measure prefrontal CTs were re-recruited after the pandemic. They subsequently completed the COVID Stress Scale (CSS) to assess stress, the Clinician Administered PTSD Scale-5 (CAPS) to diagnose PTSD, and a second sMRI scan. COVID-19 infection was self-reported. Associations between stress and symptom assessments and post-pandemic CTs, differences in CTs in PTSD vs. non-PTSD groups, and changes in pre- to post-pandemic CTs were examined. **Results**: Pre-pandemic PCL scores were positively associated with CSS scores which, in turn, were higher in the PTSD group. Thicker IFG-opercularis CTs were associated with COVID-19 infection. Post-pandemic rMFG and IFG-orbitalis CTs were positively associated with CAPS scores. rACC CTs were negatively associated with CSS scores. Pre- to post-pandemic rMFG and frontal pole CTs thickened in the PTSD group but thinned in the non-PTSD group, whereas rACC CTs thinned in the PTSD group but thickened in the non-PTSD group. **Conclusions**: These findings provide novel evidence that the COVID-19 pandemic had diverse effects involving prefrontal cortex structure, stress, and PTSD symptoms in subjects with pre-pandemic trauma history and suggest that treatments are needed to counter these diverse effects.

## 1. Introduction

The COVID-19 pandemic caused unprecedented mental health challenges, including prolonged and elevated global psychological distress [1,2]. Significant pandemic-related increases in depression, anxiety, and posttraumatic stress disorder (PTSD) in diverse populations, e.g., healthcare workers, young adults, and others, have been attributed to pandemic-related stress [3,4]. High levels of stress, anxiety, and PTSD have been linked to pandemic-related fear and lockdown [5].

Stress affects brain changes that contribute to physical and emotional adaptation to stress and improves survival [6,7]. The prefrontal cortex (PFC) regions serve as integration centers for regulation of emotional behavior, fear extinction, and decision making that is essential for stress adaptation [8]. Prolonged or intense stress can impair PFC function on emotion regulation in healthy individuals and individuals with pre-existing mental health conditions [9]. PFC dysfunction can increase vulnerability to develop abnormal responses to future stressors by exacerbating symptoms like hypervigilance and emotional dysregulation [10,11]. Deficits in emotion regulation are linked to psychiatric disorders, including, e.g., depression, anxiety, and PTSD [12,13,14,15]. From this, it might be expected that individuals with pre-existing conditions, such as previous trauma exposure, may have different responses to pandemic-related stress. Meta-analyses of patients with pre-existing mental illness suggest that these patients had significantly higher stress and psychiatric symptoms during the pandemic than the control groups [16]. A new understanding of brain regions affected by pandemic-related stress in patients who had pre-pandemic traumatic experiences or other pre-existing conditions is desirable and needed to direct clinical interventions and personalized treatment to improve mental health outcome.

Structural changes in PFC have been reported in psychiatric patients. PTSD studies indicate that CT reductions in PFC regions, which are associated with early life trauma and PTSD symptoms, can impair stress responses and emotion regulation [17,18]. Volume reductions in ventral medial PFC (vmPFC), which encompasses the rostral anterior cingulate cortex (rACC) and medial orbitofrontal cortex (mOFC), have been reported in PTSD patients [19,20]. Moreover, greater vmPFC thickness appears to be associated with better recall of fear extinction memory [21]. Recent mega-analyses from our lab suggest that chronic PTSD is associated with reduced cortical volumes in widespread emotion regulation regions, including orbital frontal, rostral middle frontal, and pars-orbitalis inferior frontal regions [22]. A longitudinal imaging study found that trauma survivors had thicker dorsolateral prefrontal (DLPFC), superior frontal (SFG), and inferior frontal (IFG) cortices 1.4 years after trauma that subsequently normalized over 5 years and which, in turn, were associated with PTSD symptoms [23]. PTSD symptoms have also been associated with post-earthquake gray matter volume (GMV) changes in the left lateral OFC [24]. Taken together, the above findings suggest that PFC plays a role in stress and PTSD after trauma exposure. Most brain structural studies of PTSD assess survivors of a single intense traumatic event or trauma occurring in early life. It would be further useful to extend the study to how PFC structure in adults is affected by prolonged stressful living conditions as were common during COVID-19 pandemic.

Early work examined changes in brain structures, which were associated with COVID-19 viral infection and pandemic-related fear [25,26]. Compared to healthy controls, reductions in cortical and subcortical volumes and frontal, temporal, and parietal cortical thicknesses had been observed in COVID-19 patients. These structural changes appear to be associated with COVID-19 symptoms and cognitive deficits [26,27,28,29,30]. Further study in COVID-19 patients reported increased GMV in PFC and other brain regions that correlated with PTSD symptoms [31]. The available literature largely focuses on COVID-19 patients with major neurological symptoms. There is limited work on associations between pandemic-related stress and brain structural changes in the cortical regions involved in stress and emotion regulation, particularly in individuals with pre-existing mental conditions.

The objective of the present study was to examine brain and psychological reactions to the COVID-19 pandemic in subjects who had suffered pre-pandemic trauma. Given that PFC contributes to stress responses, we were particularly interested in how CTs in PFC regions were affected in these subjects. We directly examined associations between COVID-19 viral infection, pandemic-related stress, PTSD symptoms, and PFC cortical thicknesses, as well as PTSD vs. non-PTSD group differences. The findings provide new insights into the impact of pandemic on brain structure, stress, and PTSD in subjects who had pre-pandemic conditions.

## 2. Materials and Methods

### 2.1. Participants and Processing

The participants were recruited from a previous trauma study conducted in our lab [32]. Briefly, trauma survivors were recruited from local hospitals immediately after a life-threatening traumatic event. They completed a post-trauma stress symptom assessment using the self-reported PTSD Checklist for DSM-V (PCL) and a brain structural MRI (sMRI) scan one year after the trauma. These survivors were recontacted after the outbreak of the COVID-19 pandemic in March 2020. We excluded survivors who (1) tested COVID-19-positive during the in-person study session, (2) had contraindications for MRI scanning, (3) were diagnosed with severe psychiatric or neurological problems, or (4) were under the influence of alcohol or substances at the time of study. This study was approved by the University Institutional Review Board. All participants provided written informed consent.

The consented subjects completed the online self-report COVID Stress Scales (CSS), a virtual PTSD diagnosis interview conducted by a clinical psychologist, and a sMRI scan after a confirmed negative COVID-19 test. Information about COVID-19 infection and treatment history was tracked by self-report. Pre-pandemic PCL scores and sMRI data were derived from the parent study.

### 2.2. Pandemic Stress Assessment

The 37-item CSS survey was used to assess stress induced by the COVID-19 pandemic, including fears to danger, contamination, economic consequences, xenophobia, compulsive checking, and traumatic stress [33]. The CSS total score and 6 subscores were calculated from (1) 6 questions about fear of danger; (2) 6 questions about socioeconomic fear; (3) 6 questions about xenophobia; (4) 6 questions about contamination fear; (5) 6 questions about traumatic stress; and (6) 7 questions about compulsive checking. The CSS total score was used to represent a cumulative measure of COVID-19-related stress during the pandemic.

### 2.3. PTSD Symptom Assessment and PTSD Diagnosis

A virtual PTSD diagnostic interview was conducted by a clinical psychologist using the Clinician-Administered PTSD Scale for DSM-V (CAPS). CAPS evaluated PTSD symptom severity across four symptom clusters: reexperiencing, avoidance, negative alterations in cognition and mood, and hyperarousal, based on symptom frequency and intensity. A diagnosis of PTSD required at least 1 reexperiencing, 1 avoidance, 2 negative mood, and 2 hyperarousal symptoms by DSM-V criteria [34]. A diagnosis of partial PTSD required at least 1 symptom in each of these symptom clusters. Partial PTSD was included because partial PTSD with impairment of social functioning commonly requires clinical intervention [35]. The CAPS total score was also calculated by summing the ratings of 20 PTSD symptoms. CAPS has high internal consistency (α = 0.88) and interrater reliability (к = 0.91), as well as good test-retest reliability (к = 0.78) [34].

Pre-pandemic stress symptoms 1 year after a pre-pandemic life-threatening traumatic event were reflected by the self-reported PCL scores obtained from the previous study. The PCL survey evaluates the severity of 20 symptoms for reexperiencing, avoidance, negative alterations in cognition and mood, and hyperarousal symptom clusters. These were the same symptoms assessed by CAPS. Each item was rated on a 5-point Likert scale (0 = Not at all, 4 = Extremely) with the total score ranging from 0 to 80. PCL survey has strong internal consistency (α = 0.94) and test–retest reliability (r = 0.82) [36].

### 2.4. Structural MRI Acquisition, Processing, and Cortical Thickness Measures

All participants were scanned using a 3T General Electric Signa HDx MRI scanner (GE Healthcare, Chicago, IL, USA). A high-resolution T1-weighted sMRI image was obtained using a previously validated 3D FSPGR structural MRI image protocol (TR = 7.836 ms, TE = 2.976 ms, FA = 9°, NEX = 1, field of view = 256 × 256 mm, matrix = 256 × 256, slice thickness = 1 mm, voxel dimensions = 1 × 1 × 1 mm^3^, 164 contiguous axial slices) [32]. sMRI image quality was checked by a trained MRI team member. The subjects with blurred sMRI images caused by head motion during the scan were excluded from sMRI analyses. The sMRI images were also reviewed by an experienced radiologist to identify clinical abnormalities.

Brain sMRI scans were processed using FreeSurfer software (Version 7) (https://surfer.nmr.mgh.harvard.edu) (accessed on 23 March 2025). Using the automated FreeSurfer processing stream, the surface-based CT map was built from ~150,000 vertex measures from each hemisphere at which CT was defined as the distance from the gray matter pial surface to the gray–white matter border [37]. An experienced MRI team member who was blinded to psychological assessments visually inspected FreeSurfer-created cortical gray matter borders on each MRI slice for all subjects and made necessary corrections.

Based on mega-analyses of PFC cortical changes related to PTSD ([22], see Introduction), PFC regions of interest (ROIs) included the superior-, middle-, and inferior-frontal, orbital, anterior cingulate, and frontal pole regions. The mean CT for each ROI in each hemisphere was defined by FreeSurfer software.

### 2.5. Statistics/Analysis

#### 2.5.1. PTSD vs. Non-PTSD Group Comparison Analyses

The number of participants having at least one positive COVID-19 test after the pandemic was compared for PTSD vs. non-PTSD groups using a chi-square test to assess if the COVID-19 infection differed between the two groups. The CSS total score and subscores were compared using ANCOVA analyses to assess potential differences in pandemic-related stress across these groups. ANCOVA analyses were also used to evaluate the effects of COVID-19 infection and post-pandemic PTSD diagnosis on post-pandemic CTs, as well as investigate how pre-pandemic PCL scores contributed to these effects.

#### 2.5.2. Correlation Analyses

Partial correlation analyses were used to examine relationships between pre-pandemic PCL scores and pandemic CSS total scores and subscores. Partial correlation analyses were also used to assess relationships between CSS scores, CAPS scores, and prefrontal CTs.

#### 2.5.3. Repeated Measures ANCOVA Analysis

In subjects with both pre-pandemic and post-pandemic sMRI data, post-pandemic CT changes in PFC regions were compared to pre-pandemic measures. PTSD*time interactions and COVID-19 infection*time interactions on CT changes were tested using the repeated measures ANCOVA. The pre- to post-pandemic CT differences in individual subjects and percentages of subjects contributing to the results of the repeated measures analysis were also calculated.

Age, sex, time between pandemic onset to survey and sMRI data collections, and days between two sMRI scans were included as covariant in relevant analyses. Given that depression is commonly comorbid with PTSD [22,38], and that social support is a resilience factor for trauma recovery [39], we assessed pandemic impacts on depression and general anxiety mood using the COIVD-related Impacts on Mood Survey. Similarly, social support during the pandemic was assessed using the Perceived Social Support Scale (see Appendix A). These measures did not differ in PTSD vs. non-PTSD groups and were not significantly correlated to CTs in PFC regions. Thus, we did not include these factors in the analyses presented in this study. Statistical analyses were performed using SPSS version 29 (IBM Corp., Armonk, NY, USA). The data are reported as mean ± SD, with *p* < 0.05 set as the significant level. Correlation analysis figures were created with SPSS, and Excel charts were used for bar graphs, pre- to post-pandemic line charts, and figure assembly.

## 3. Results

### 3.1. Demographics

Fifty-one subjects (37 females, 14 males) who had experienced a life-threatening traumatic event were recruited from our previous study. Five subjects withdrew from sMRI scanning and four were excluded due to blurred sMRI images. Thus, 42 subjects were included in sMRI analyses.

The data regarding age, race, time between pandemic onset (3/01/2020 in US) and the survey, sMRI, days between two sMRI scans, and the numbers of subjects with PTSD diagnosis and COVID-19 infection are summarized in Table 1. Forty-four subjects completed the post-pandemic PTSD diagnosis interview. Using CAPS for DSM-V criteria, 16 subjects were diagnosed with PTSD, whereas 28 were diagnosed as non-PTSD. Forty-nine subjects had COVID-19 test records. Nineteen reported at least one positive COVID-19 test and seven subjects received treatment for COVID-19 infection. No participants were being treated for COVID-19 symptoms at the time of this study. There was no significant difference in the number of subjects who had at least one positive COVID-19 test in the PTSD vs. non-PTSD group (Table 1).

### 3.2. Correlation Analysis of Pre-Pandemic PCL Scores and Pandemic CSS Scores

**Figure 1:**
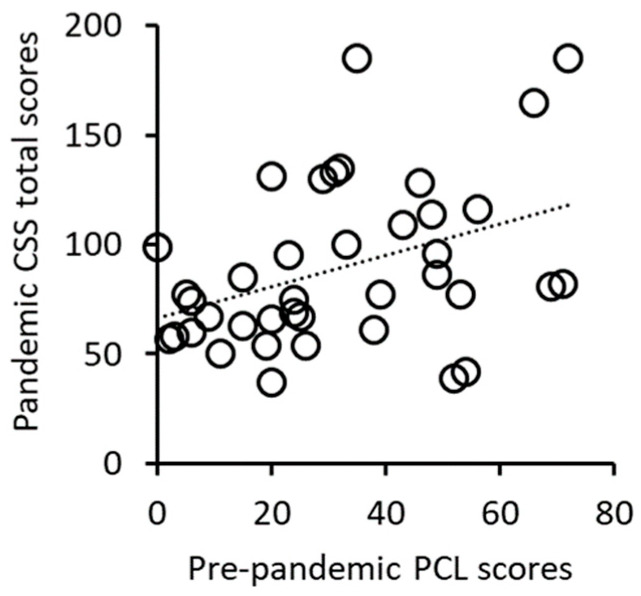
Positive correlation between pre-pandemic PCL scores and pandemic CSS total scores.

The pre-pandemic PCL scores were significantly positively correlated with the pandemic CSS total scores (r = 0.429, *p* = 0.009, df = 34, Figure 1, Table 2). Significant positive relationships were also seen between the pre-pandemic PCL scores and CSS subscores for danger, economic, traumatic stress, and compulsive checking (Table 2). These results suggest that subjects with more severe pre-pandemic PTSD symptoms from prior trauma experienced greater pandemic-related stress.

### 3.3. Analyses of Pandemic CSS Scores in Post-Pandemic PTSD vs. Non-PTSD Subjects

The pandemic CSS total scores did not significantly differ in PTSD vs. non-PTSD group; however, the CSS subscores for danger fear and traumatic stress were significantly higher in the PTSD group than non-PTSD group (Table 3). The other CSS subscores did not differ between two groups (Table 3). These results suggest that the post-pandemic PTSD subjects experienced greater pandemic-related stress than non-PTSD subjects with regard to stress related to fear about COVID-19 danger and traumatic stress.

### 3.4. Correlation Analyses of Post-Pandemic Prefrontal Cortical Thicknesses, PTSD Symptoms, and COVID-19-Related Stress

**Figure 2:**
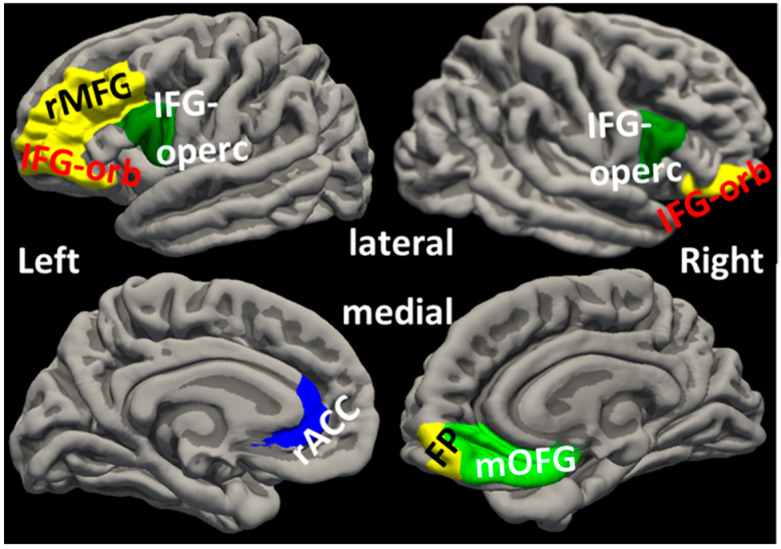
Locations of cortical regions. FP: frontal pole; IFG-orb: pars orbitalis of inferior frontal gyrus; IFG-operc: pars opercularis of inferior frontal gyrus; mOFG: medial orbital frontal gyrus; rACC: rostral anterior cingulate cortex; rMFG: rostral middle frontal gyrus.

**Figure 3:**
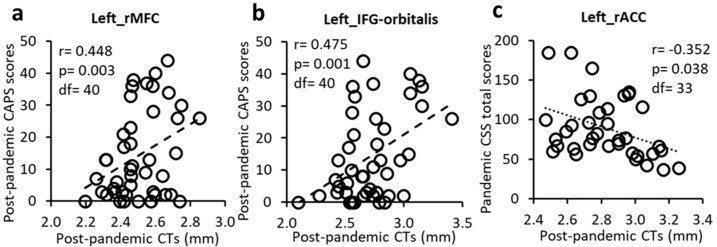
Post-pandemic CTs positively correlated with post-pandemic CAPS scores in the left rMFG (**a**) and IFG-orbitalis (**b**) but negatively correlated with CSS total scores in the left rACC (**c**).

The post-pandemic CTs in the left rostral middle frontal gyrus (rMFG) and the left pars orbitalis of the inferior frontal gyrus (IFG-orbitalis) were significantly positively associated with CAPS scores (Figure 2 and Figure 3a,b). These results suggest that thicker post-pandemic CTs in these regions were positively related to worse post-pandemic PTSD symptoms.

CT in the left rACC was significantly negatively associated with pandemic CSS total score (Figure 2 and Figure 3c).

Together, these findings suggest that CTs of lateral PFC and rACC regions played roles, respectively, in severity of post-pandemic PTSD symptoms and pandemic stress.

### 3.5. Effects of COVID-19 Infection and PTSD on Post-Pandemic Prefrontal Cortical Thicknesses

**Figure 4:**
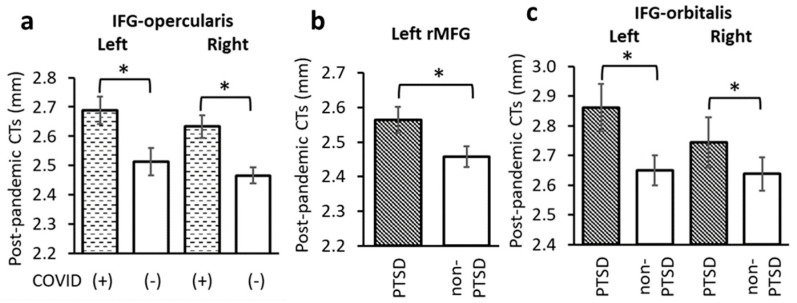
(**a**) CTs in the left and right IFG-opercularis were significantly thicker in subjects with a history of COVID-19 infection. CTs in (**b**) the left rMFG and (**c**) left and right IFG-orbitalis were significantly thicker in the PTSD than non-PTSD groups. *: *p* < 0.05 as significant level, controlled for age, sex and time between pandemic onset and sMRI data collection.

COVID-19 infection significantly impacted IFG-opercularis CTs. Specifically, subjects with a history of a positive COVID-19 test had thicker post-pandemic CTs in the left and right IFG-opercularis than subjects who did not have a positive test history (left: F = 6.106, *p* = 0.018; right: F = 8.396, *p* = 0.006, Figure 2 and Figure 4a). These results remained significant when controlled for pre-pandemic PCL scores (left: F = 6.184, *p* = 0.020; right: F = 7.558, *p* = 0.011).

The PTSD group had significantly thicker post-pandemic CTs than the non-PTSD group in the left rMFG (F = 4.596, *p* = 0.040, Figure 2 and Figure 4b). This result remained significant when controlled for pre-pandemic PCL scores (F = 5.456, *p* = 0.027). Compared to the non-PTSD group, the PTSD group also had significantly thicker post-pandemic CTs in the left and right IFG-orbitalis (left: F = 4.248, *p* = 0.049; right: F = 4.813, *p* = 0.037, Figure 2 and Figure 4c).

The effects of pre-pandemic PCL scores and COVID-19*PTSD interaction were not significant for any prefrontal CTs.

Taken together, the above results suggest that COVID-19 infection contributed to thickening CTs in IFG-opercularis. In addition, post-pandemic PTSD diagnosis appeared related to CT thickening in rMFG and IFG-orbitalis.

### 3.6. Changes in Prefrontal Cortical Thickness from Pre- to Post-Pandemic Time and Associations with PTSD and COVID-19 Infection

**Figure 5:**
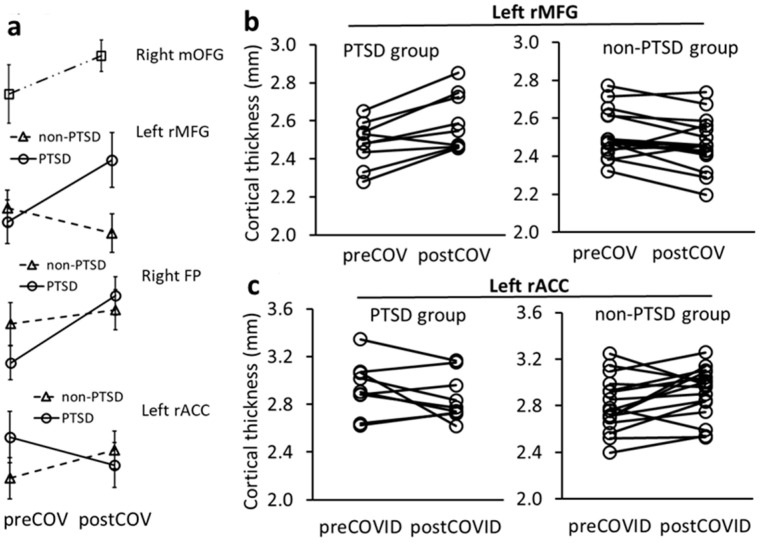
Repeated measures ANCOVA analysis for CT changes in PFC in PTSD and non-PTSD subjects over pre- to post-pandemic times. (**a**) Mean (±SD) CT changes over time in the right mOFG across all subjects, and in the left rMFG, right FP and left rACC in PTSD and non-PTSD groups. (**b**) Over time, CT in the left rMFG increased in 88.9% of subjects in the PTSD group but decreased in 81.2% of non-PTSD subjects. (**c**) Over time, CT in the left rACC decreased in 55.6% of subjects in the PTSD group but increased in 75% of non-PTSD subjects.

An exploratory analysis of CT changes over time from before to after the pandemic was conducted on a subgroup of 9 subjects in the PTSD group and 16 subjects in the non-PTSD group who had undergone pre- and post-pandemic sMRI scans. Repeated measures ANCOVA suggested that post-pandemic CT in the right mOFG was thicker than before the pandemic for all subjects (time effect F = 8.038, *p* = 0.012, Figure 2 and Figure 5a).

PTSD*time interactions in CT changes in the left rMFG and right frontal pole (FP) were significant and indicated that CTs thickened in the PTSD group and thinned in the non-PTSD group from before to after the pandemic (F = 11.984, *p* = 0.003; F = 5.179, *p* = 0.037, respectively, Figure 2 and Figure 5a). These group findings were further supported by individual subject results that 8 out of 9 (88.9%) PTSD subjects had thickening rMFG changes, whereas 13 of 16 (81.2%) non-PTSD subjects had thinning rMFG changes (Figure 5b).

PTSD*time interaction was also significant for CT change in the left rACC and indicated that CT in the left rACC thinned in the PTSD group and thickened in the non-PTSD group (F = 5.568, *p* = 0.031, Figure 2 and Figure 5a). This finding was supported at the individual subject level by 5 of 9 (55.6%) PTSD subjects with left rACC thinning and 12 of 16 (75%) non-PTSD subjects with left rACC thickening (Figure 5c).

COVID-19 infection*time interaction did not have a significant effect on pre- to post-pandemic CT change in any PFC regions. Pre-pandemic CTs in the PFC regions did not differ between the PTSD and non-PTSD groups.

Taken together, the above findings suggest that pre- to post-pandemic prefrontal CT changes differed between those pre-pandemic trauma survivors who had PTSD and those who did not. It also appears that different PFC regions, including rMFG, FP, and rACC, underwent different pre- to post-pandemic thickness effects.

## 4. Discussion

The present study suggests that higher pre-pandemic PTSD symptoms in pre-pandemic trauma survivors were positively associated with more severe COVID-19-related stress during the pandemic. Positive associations were observed between post-pandemic CAPS scores and post-pandemic CTs in the left rMFG and IFG-orbitalis. CTs in the left rMFG and the left and right IFG-orbitalis were also significantly thicker in subjects who were diagnosed with post-pandemic PTSD. A negative association was found between the pandemic CSS score and post-pandemic CT in the left rACC. Post-pandemic CT thickening in the left and right IFG-opercularis was associated with COVID-19 infection. Analysis of pre- to post-pandemic CT changes across all subjects suggested right mOFG CT thickened. PTSD*time interactions indicated significant pre- to post-pandemic CT changes, with the PTSD group showing increased CTs in the left rMFG and right FP and decreased CTs in the left rACC. In contrast, the non-PTSD group had opposite pre- to post-pandemic CT changes in these regions. These results suggest that the COVID-19 pandemic affected stress responses and prefrontal CTs differently between those pre-pandemic trauma survivors who had PTSD and those who did not.

### 4.1. PFC Involvement in Stress and PTSD

The present results suggest that the PFC regions contributed to the COVID-19 pandemic stress. PFC contains high-order executive processing centers that play key roles in stress, emotion regulation, cognitive control, attention, and working memory [9]. Prefrontal cortical regions are essential for daily adaptations to environmental and physical demands. Stress studies have demonstrated that abnormal levels of stress neurotransmitters and glucocorticoids in response to intense or chronic stress affect PFC function and/or structure [17]. Prolonged or intense stress can induce PFC executive function, emotion regulation, and structural impairment [40]. Patients with mood and anxiety disorders commonly exhibit emotional dysregulation [41]. Subjects in the present study had experienced a life-threatening traumatic event prior to the pandemic. Those who subsequently reported severe PTSD symptoms may have developed PFC dysfunctions that impaired responses to the pandemic. Our results point to a positive association between pre-pandemic PCL scores and pandemic CSS scores. COVID-19-related stress, including fear related to virus danger and traumatic stress, were greater in the post-pandemic PTSD group than non-PTSD group. This suggests that trauma survivors with more severe pre-pandemic PTSD symptoms may have had deficits in stress control, negative attention bias, and emotional dysregulation. Consequently, they may have been more vulnerable to pandemic-related stress and abnormal PFC emotion processing and regulation in response to pandemic threats.

As part of a response to prolonged pandemic stress, PFC likely increased stress management to cope with heightened emotional and cognitive demands [42]. PFC changes occur in response to behavioral and psychological interventions in psychiatric patients [43]. Volume and thickness reductions in DLPFC in PTSD patients are associated with dysfunction in negative emotion regulation [41,44,45]. Alternatively, thicker frontal CTs have been reported to positively associate with PTSD symptom severity [46]. In a trauma longitudinal subway disaster study, trauma survivors who did not have pre-existing trauma experience had thicker DLPFC CTs at 1.4 years after trauma than trauma-free controls, and DLPFC CTs subsequently normalized over 5 years [23]. The present findings suggest that CT in the left rMFG, a part of DLPFC, was thicker in the post-pandemic PTSD group than non-PTSD group. This CT thickening was positively associated with PTSD symptoms. There are notable differences between the present study and a subway disaster study. The subjects in present study had a pre-pandemic life-threatening trauma experience and the pandemic was their further stress, whereas subjects in the subway disaster study did not have a trauma experience before the disaster. This difference may account for different CT effects. In addition, the subway disaster study did not address whether CT normalization in DLPFC occurred in PTSD and non-PTSD survivors. From our results, further work would be useful to longitudinally track if subjects who were diagnosed with post-pandemic PTSD can undergo recovery from PTSD and reversible PFC thickness changes.

rMFG is thought to play key roles in stress and emotion regulation by inhibitory control over, e.g., low-order brain regions and stress perception and appraisal [47]. Increased CTs in the left and right rMFG have been reported to be positively associated with perceived stress [48]. Abnormal rMFG activity has been linked to increased stress perception and depressive symptoms [9]. In the present study, 88.9% of subjects with post-pandemic PTSD had thicker CT in the left rMFG, whereas 81.2% of post-pandemic non-PTSD subjects had thinner left rMFG. These findings suggest that cortical structural alterations in the left rMFG may have contributed to pandemic-related stress that potentially contributed to PTSD.

We observed greater left and right IFG-orbitalis CTs in post-pandemic PTSD than non-PTSD subjects. The left IFG-orbitalis CTs was positively associated with CAPS scores. IFG is thought to play important roles in executive tasks, attention, and memory [49]. IFG is divided in a rostral-caudal direction into the pars orbitalis, pars triangularis, and pars opercularis [50]. IFG-orbitalis is located around Brodmann area 47 that is involved in processing of disgusted, angry, and fearful facial expressions [51]. IFG-orbitalis has been reported to contribute to recognition of emotions and to inhibition of adverse emotion [52,53]. This region may also play a role in integrating emotional recognition with executive prediction and interact with the orbitofrontal cortex in behavioral adaptations [54]. Other work has reported that trauma exposure can significantly impact IFG functions [55]. Previous research suggests that abnormal IFG activation contributed to impaired fear inhibition in trauma survivors with PTSD [56]. In the present study, larger CTs in the left and right IFG-orbitalis were associated with post-pandemic PTSD symptoms. These findings raise the possibility that structural changes in IFG-orbitalis may contribute to brain maladaptations to heightened emotional and cognitive demands from the pandemic.

CT in the left rACC was negatively associated with pandemic-related stress. Analysis of pre- to post-pandemic CT changes suggested CT in the left rACC decreased over time in PTSD subjects but increased over time in non-PTSD subjects, thus, suggesting that left rACC CT underwent different pandemic changes in PTSD vs. non-PTSD subjects. rACC has extensive connections with medial PFC and plays a critical role in emotional regulation and stress control [57]. Previous work reports that thinner rACC is linked to emotion processing disruption in PTSD patients [58]. A study on earthquake survivors reported that thinner left and right rACC CTs in PTSD survivors were negatively correlated with CAPS scores [59]. Taken together, our findings of a negative association between post-pandemic CT in the left rACC and pandemic-related stress, as well as pre- to post-pandemic left rACC thickening in non-PTSD subjects suggest that thicker left rACC CT may be associated with reduced stress and PTSD symptoms. In contrast, rACC CT thinning may be associated with vulnerability to stress.

### 4.2. PFC Involvement in COVID-19 Viral Infection

About 70% of COVID-19 patients report loss of smell or taste as their initial symptoms [60,61]. In the present study, thicker IFG-opercularis CT was associated with COVID-19 infection. The human gustatory cortex, which perceives and distinguishes different tastes, is located in IFG-opercularis and anterior temporal regions [62]. COVID-19 studies suggest the COVID-19 virus can attack the central nervous system through the olfactory pathway or blood–brain barrier, and, thus, cause loss of taste, smell and other neurological symptoms [63]. A recent study of long COVID-19 symptoms reported decreased odor discrimination that was associated with activation in the right frontal pole and superior frontal gyrus, but did not detect gustatory deficits [64]. Current views on COVID-19-induced taste loss focus on dysfunctions in tongue taste buds as a consequence of viral attack on taste receptors [65]. The present findings suggest that thicker left and right IFG-opercularis CTs were associated with COVID-19 infection and provide a first suggestion that the COVID-19 virus may affect gustatory cortex structure as well as taste receptors.

COVID-19-related brain changes remain controversial and likely vary due to COVID-19 symptom severity, duration, and other factors [66,67]. Autopsies of COVID-19 patients reveal diverse cellular changes in cerebral cortex and other brain structures, including, e.g., increased synaptic connections, activation of microglia and astrocytes, and neuroinflammation [68]. The impacts of COVID-19 virus on the human brain are complex and its invasion pathways and mechanisms to destroy brain structures are far from being completely understood. The possibilities include immune response and proinflammatory reactions in neurons and glia, cellular infection by binding to cell membrane receptors, and viral RNA replication, transcription, and spread. COVID-19 virus-caused lung and vascular lesions can induce hypoxia and neuron hypoxic injury. In addition, the COVID-19 virus may infect intestinal peripheral neurons and choroidal plexus epithelial cells as an alternative pathway to invade the brain [63].

The current finding, that the left and right IFG-opercularis CT thickening was associated with COVID-19 viral infection, appears inconsistent with findings from the UK Biobank study where post-pandemic CTs in the olfactory cortex, including the orbitofrontal cortex, were thinner in mild COVID-19 patients compared to pre-pandemic baselines [25]. The reasons for this apparent difference remain unclear but may involve study differences. For example, subject ages ranged from 19 to 55 years in the present study and from 51 to 81 years in UK Biobank study. It is well known that age affects cortical thickness [69]; therefore, differences in subject ages and other factors may have contributed to differences in study results.

### 4.3. Limitations

This study has limitations. First, only individuals who experienced a traumatic event before the pandemic were studied. Second, the findings do not provide direct links between CT changes and COVID-19 symptoms. Third, the data were collected approximately 1.5–2 years after the outbreak of the pandemic and, thus, do not address different potential stages of pandemic-related changes. Fourth, the study focused on pandemic-related stress and PTSD symptoms and did not assess further cognitive functions. Fifth, analyses followed a standard statistical approach with controlling the probability of Type I error for all *p*-value calculations, while the probability of Type II error was not explicitly considered. Had the pandemic been expected, a more formally designed experiment, including a formal sample size calculation, could have been planned. A potential consequence of the study’s small sample size is an increased probability of Type II error. As a consequence, we may have missed some Type II false negative errors. Thus, from a Type I error perspective, our results are statistically conservative; however, from a Type II error perspective, we may have missed further relevant findings. Finally, the small sample size did not allow for detailed analyses of pre- to post-pandemic CT change dynamics.

## 5. Conclusions

The present findings provide original evidence that the COVID-19 pandemic had demonstrable effects on prefrontal cortical structure, stress, and PTSD symptoms in subjects who had experienced pre-pandemic trauma. There is conjecture that people who had pre-existing mental health challenges might have been more vulnerable to brain and mental-health impacts caused by the pandemic. The present findings suggest that treatments are needed to counter these diverse effects.

## Figures and Tables

**Table 1:** Demographic data.

	Mean ± SD or N
Age (year, (range))	35.1 ± 11.3 (19–55)
Time between (days)	
pandemic onset to survey	794 ± 233
pandemic onset to sMRI	700 ± 261
Days between 2 sMRI scans	1299 ± 289
Race (N)	
White	19
Black	27
Asian	1
Mixed	2
Unknown	2
Post-pandemic PTSD (N/total)	16/44
Positive COVID-19 test (N/total)	19/49
	PTSD:non-PTSD
positive COVID-19 test	8:10
Negative COVID-19 test	7:17
*χ*^2^, *p*	1.046, 0.307

**Table 2:** Relationships between pre-pandemic PCL scores and pandemic CSS scores.

Relationships	Mean ± SD	r, *p* (df = 34)
Pre-pandemic PCL score	31.9 ± 20.3	
CSS-danger	16.9 ± 7.9	0.349, 0.037 *
CSS-Economic	15.6 ± 7.4	0.376, 0.024 *
CSS-Xenophobia	11.4 ± 7.0	0.276, 0.104
CSS-Contamination	13.9 ± 6.8	0.272, 0.109
CSS-Traumatic	13.0 ± 7.0	0.520, 0.001 *
CSS-Compulsive	18.2 ± 8.0	0.365, 0.029 *
CSS total score	88.9 ± 36.7	0.429, 0.009 *

*: *p* < 0.05 as significant level, controlled for age, sex, and time between pandemic onset and survey data collection.

**Table 3:** Comparisons of pandemic CSS scores in post-pandemic PTSD vs. non-PTSD groups.

	PTSD Group	Non-PTSD Group	*Group Comparison (f, p)*
CSS-Danger	19.5 ± 9.2	14.6 ± 6.6	4.217, 0.048 *
CSS-Economic	17.5 ± 8.0	13.8 ± 6.7	2.509, 0.122
CSS-Xenophobia	11.9 ± 7.2	9.9 ± 5.7	2.449, 0.127
CSS-Contamination	14.4 ± 8.0	12.7 ± 5.5	1.202, 0.280
CSS-Traumatic	15.3 ± 7.8	11.1 ± 5.5	4.732, 0.036 *
CSS-Compulsive	19.3 ± 8.1	17.0 ± 7.7	0.671, 0.418
CSS total score	97.9 ± 38.3	79.2 ± 27.4	3.638, 0.065

*: *p* < 0.05 as significant level, controlled for age, sex and time between pandemic onset and survey data collection.

## Data Availability

The original contributions presented in this study are included in the article material. Further inquiries can be directed to the corresponding author.

## Appendix A

### Appendix A.1. Assessments

The COVID-19 pandemic’s impacts on depression and anxiety mood were assessed using the COIVD-related Impacts on Mood Survey (https://www.phenxtoolkit.org/toolkit_content/PDF/UFl_Coing_with_COVID19_Mood.pdf) (assessed on 23 March 2025), which was adapted from Neuro-QOL Item Bank v1.0—Anxiety and Depression—Short Forms. The participants were requested to recall eight depression symptoms and eight general anxiety symptoms before the COVID-19 pandemic and evaluate if each symptom became worse, remained the same, or became better after pandemic, rating from 1 to 5. Lower scores reflected more severe depression and anxiety symptoms after the pandemic.

The social support during the pandemic was assessed using the modified Perceived Social Support Scale (PSS). The PSS questionnaire includes four items about social support from family and friends during the pandemic. A higher score means stronger social support.

Univariate ANCOVA analyses were used to compare the pandemic impacts on mood and social support in post-pandemic PTSD vs. non-PTSD groups. The correlations between these assessments and prefrontal CTs were conducted using partial correlation analysis.

### Appendix A.2. Results

The PSS score did not differ in post-pandemic PTSD vs. non-PTSD groups. The pandemic’s impacts on depression and anxiety mood also did not reach significant difference between two groups.

**Table A1:** Comparisons of pandemic PSS and impacts on mood changes between post-pandemic PTSD vs. non-PTSD groups [38,39].

	PTSD Group	Non-PTSD Group	Comparison (f, *p*)
PSS	18.2 ± 7.3	21.1 ± 7.7	1.632, 0.210
Depression mood	17.0 ± 7.5	23.6 ± 8.8	3.599, 0.067
Anxiety mood	16.7 ± 9.8	22.2 ± 6.8	3.888, 0.057

There were no significant correlations between post-pandemic prefrontal CTs, PSS scores, and pandemic impacts on depression and anxiety mood.

**Table A2:** Correlations between post-pandemic prefrontal CTs, PSS scores, and pandemic impacts on depression and anxiety mood [38,39].

PFC Cortical Thicknesses		PSS Score	Depression Mood	Anxiety Mood
L_caudalanteriorcingulate	Correlation	0.189	−0.344	−0.258
	*p*	0.277	0.054	0.141
	df	33	30	32
L_caudalmiddlefrontal	Correlation	0.078	0.138	0.017
	*p*	0.655	0.451	0.922
	df	33	30	32
L_lateralorbitofrontal	Correlation	0.081	0.049	−0.116
	*p*	0.643	0.792	0.513
	df	33	30	32
L_medialorbitofrontal	Correlation	0.134	−0.228	−0.201
	*p*	0.444	0.21	0.255
	df	33	30	32
L_parsopercularis	Correlation	0.018	0.012	−0.081
	*p*	0.918	0.946	0.651
	df	33	30	32
L_parsorbitalis	Correlation	−0.142	−0.177	−0.21
	*p*	0.416	0.332	0.232
	df	33	30	32
L_parstriangularis	Correlation	−0.109	0.004	−0.169
	*p*	0.535	0.983	0.338
	df	33	30	32
L_rostralanteriorcingulate	Correlation	0.27	−0.04	−0.061
	*p*	0.117	0.828	0.734
	df	33	30	32
L_rostralmiddlefrontal	Correlation	−0.084	−0.008	−0.084
	*p*	0.632	0.964	0.638
	df	33	30	32
L_superiorfrontal	Correlation	0.063	−0.007	−0.067
	*p*	0.721	0.971	0.705
	df	33	30	32
L_frontalpole	Correlation	−0.201	−0.111	−0.088
	*p*	0.246	0.545	0.62
	df	33	30	32
R_caudalanteriorcingulate	Correlation	−0.19	−0.013	−0.001
	*p*	0.275	0.945	0.994
	df	33	30	32
R_caudalmiddlefrontal	Correlation	0.232	0.204	0.079
	*p*	0.179	0.264	0.659
	df	33	30	32
R_lateralorbitofrontal	Correlation	0.099	0.066	−0.02
	*p*	0.57	0.721	0.908
	df	33	30	32
R_medialorbitofrontal	Correlation	−0.085	0.195	−0.01
	*p*	0.627	0.285	0.955
	df	33	30	32
R_parsopercularis	Correlation	0.073	−0.16	−0.168
	*p*	0.679	0.383	0.343
	df	33	30	32
R_parsorbitalis	Correlation	−0.284	0.059	−0.103
	*p*	0.099	0.749	0.563
	df	33	30	32
R_parstriangularis	Correlation	−0.008	−0.121	−0.161
	*p*	0.965	0.509	0.363
	df	33	30	32
R_rostralanteriorcingulate	Correlation	−0.037	−0.02	−0.143
	*p*	0.831	0.912	0.419
	df	33	30	32
R_rostralmiddlefrontal	Correlation	−0.083	−0.02	−0.158
	*p*	0.634	0.915	0.373
	df	33	30	32
R_superiorfrontal	Correlation	−0.001	0.086	−0.037
	*p*	0.995	0.64	0.834
	df	33	30	32
R_frontalpole	Correlation	0.001	−0.001	0.026
	*p*	0.994	0.997	0.882
	df	33	30	32
